# Animal-derived collagen and Islamic religious prescriptions: a systematic review

**DOI:** 10.1007/s10067-026-08247-z

**Published:** 2026-06-25

**Authors:** Raimondo Leone, Alberto Migliore, Walter Ricciardi

**Affiliations:** 1 Department of Life Sciences and Public Health, Università Cattolica del Sacro Cuore, Rome, Italy; 2 U.O.S. of Rheumatology, Ospedale San Pietro Fatebenefratelli, Rome, Italy

**Keywords:** Collagen, Connective tissue, Halal, Islam, Pharmaceutical preparations, Religion and medicine

## Abstract

Collagen, predominantly porcine (~ 41% of global production), is a ubiquitous pharmaceutical, cosmetic, and biomedical excipient. For the estimated 1.9 billion Muslim patients worldwide, this raises a recurrent concern with potential implications for medication adherence and patient counselling, including in rheumatology. This PRISMA 2020-compliant systematic review synthesises 56 studies retrieved from five databases (initial yield 1035 records), addressing four domains: Islamic jurisprudential classification by source, validated analytical authentication, halal-compliant alternatives, and international regulatory frameworks. Analytical studies were appraised with a modified QUADAS-2, jurisprudential sources with an internal-consistency checklist, certainty of evidence with a GRADE-informed framework. Across mainstream Sunni jurisprudence, porcine collagen is classified as prohibited (haram) regardless of industrial processing, with high inter-school agreement and convergent classical and contemporary fatwa support; bovine collagen is conditionally permissible subject to ritual (zabiha) slaughter and certified traceability; fish collagen is broadly accepted and is the most commercially mature alternative. Quantitative PCR (LOD ≈0.01%) offers the best balance of sensitivity and validation for native matrices, while LC–MS/MS proteomics is preferable for extensively hydrolysed products; orthogonal testing is advisable where stakes are high. Recombinant collagen offers the highest theoretical halal assurance but is constrained by cost, regulatory translation, and substrate certification. International harmonisation of halal pharmaceutical standards comparable to Malaysia’s MS 2424:2019 is identified as a regulatory gap. Findings are most directly relevant to pharmacists, regulators, and clinicians serving Muslim populations; direct rheumatology-specific evidence remains limited and warrants dedicated study.

**Supplementary Information:**

The online version contains supplementary material available at 10.1007/s10067-026-08247-z.

## Introduction

Collagen is the most abundant structural protein in vertebrates (~30% of total body protein mass), extracted from skin, bones, tendons, and cartilage for use in pharmaceutical excipients, wound dressings, haemostatic agents, cosmetics, and tissue-engineering scaffolds [1, 2]. Approximately 41% of global commercial collagen and gelatin production originates from porcine sources [3, 4], creating a recurrent conflict with Islamic dietary law (Shari'a), which classifies swine and their derivatives as prohibited.

An estimated 1.9 billion Muslims (about 24% of the world population) may encounter this conflict when prescribed or purchasing collagen-containing pharmaceuticals, frequently in the absence of explicit labelling of animal origin [5]. Incomplete disclosure has been associated with self-initiated non-adherence to prescribed medications in Muslim-majority settings [6]. Leone et al. have shown that concern over animal-derived biological substances is one measurable driver of vaccination hesitancy in religious communities [7], while Leone has argued that the socio-cultural and religious environment shapes health decisions in ways that purely biomedical frameworks may not fully capture [8].

To our knowledge, no prior review has synthesised this body of literature in full compliance with PRISMA 2020 [9] while integrating jurisprudential, analytical, and regulatory evidence streams. The present review aims to fill this gap and to provide clinicians, pharmacists, and policy makers with a transparent, evidence-graded synthesis of the available literature on halal collagen.

## Objectives

To synthesise evidence on (1) Islamic jurisprudential classification of collagen by animal source, distinguishing between scholarly consensus, majority opinion, and regulatory practice; (2) validated analytical methods for halal authentication, with explicit attention to specificity, validation status, and applicability across product matrices; (3) halal-compliant alternative collagen sources, distinguishing technical feasibility, commercial scalability, regulatory approval, and economic practicality; and (4) international regulatory frameworks for halal pharmaceuticals, with cautious extrapolation to potential implications for rheumatology practice.

## Methods

### Protocol and registration

The review was conducted and reported in accordance with the PRISMA 2020 statement [9]. The review was not prospectively registered in a public systematic-review registry such as PROSPERO. We acknowledge that prospective registration represents methodological best practice; the protocol, search logs, screening decisions, data-extraction matrices, and risk-of-bias judgments are nevertheless documented and are available from the corresponding author on reasonable request, to support the reproducibility of the synthesis.

### Information sources and search strategy

Five bibliographic databases were searched between 1 February and 31 March 2026: PubMed/MEDLINE, Scopus, ScienceDirect, Web of Science Core Collection, and Google Scholar (first 200 records by relevance). The reference lists of included studies and of three recent reviews were hand-searched. Records were managed in EndNote 21 and duplicates removed with a two-step procedure (automated identifier matching followed by manual review of near-duplicate titles).

The full database-specific search strings, including controlled-vocabulary terms (MeSH for PubMed; Emtree for Scopus), Boolean operators, field tags, and applied filters, are reported in Table 1 and reproduced verbatim in Supplementary Material S2. Searches were limited to records published between 1 January 2000 and 31 March 2026; no methodological filters were applied at the search stage.

**Table 1:** Database-specific search strategy (verbatim Boolean strings, filters, and date range)

Data base	Search string (verbatim)	Filters
PubMed/MED LINE	(“Collagen”[MeSH] OR “Gelatin”[MeSH] OR collagen[tiab] OR gelatin*[tiab]) AND (“Islam”[MeSH] OR halal[tiab] OR haram[tiab] OR “Islamic law”[tiab] OR shari'a[tiab] OR istihalah[tiab] OR darurah[tiab] OR “religious dietary law”[tiab]) AND (porcine[tiab] OR pork[tiab] OR swine[MeSH] OR bovine[tiab] OR fish[tiab] OR marine[tiab] OR recombinant[tiab] OR authentication[tiab] OR PCR[tiab] OR ELISA[tiab] OR “mass spectrometry”[tiab])	English; 2000–2026; humans not enforced
Scopus	TITLE-ABS-KEY((collagen OR gelatin*) AND (halal OR haram OR islam* OR shari'a OR istihalah OR darurah) AND (porcine OR pork OR swine OR bovine OR fish OR marine OR recombinant OR authentication OR PCR OR ELISA OR “mass spectrometry”)) AND PUBYEAR > 1999 AND LANGUAGE(english)	Document type: article, review, conference paper (full text)
Science Direct	(collagen OR gelatin) AND (halal OR islam* OR “porcine detection” OR “species authentication”) AND (pharmaceutical OR cosmetic OR capsule OR excipient)	Research and review articles; 2000–2026
Web of Science	TS = ((collagen OR gelatin) AND (halal OR haram OR islam* OR shari'a OR istihalah OR darurah) AND (porcine OR bovine OR fish OR marine OR recombinant OR authentication OR PCR OR ELISA OR “mass spectrometry”))	Article, review; English; 2000–2026; SCI-E and SSCI
Google Scholar	(collagen OR gelatin) AND (halal OR “islamic dietary law”) AND (porcine OR bovine OR fish OR recombinant OR authentication)	First 200 results by relevance; 2000–2026

*MeSH* Medical Subject Headings, *tiab* title/abstract field. Full search logs, including hit counts at each step, are provided in Supplementary Material S2

### Eligibility criteria

Included sources comprised peer-reviewed original research, systematic reviews, method-validation studies, and identifiable jurisprudential analyses addressing at least one of the four review domains: (a) Islamic jurisprudential analysis of collagen or gelatin; (b) analytical authentication methods for porcine DNA, peptides, or proteins; (c) halal-compliant alternative sources; or (d) regulatory or clinical implications of non-halal collagen. Inclusion was restricted to publications between 2000 and 2026 with accessible full text in English.

Excluded sources comprised conference abstracts without full text, editorial opinion pieces without primary data or structured argumentation, purely physicochemical studies without a halal component, and animal-only studies without human-relevance discussion. Duplicate publications and reports drawing on overlapping primary datasets were identified by cross-checking author lists, institutional affiliations, sample descriptions, and result tables; in such cases, the most complete (typically the most recent peer-reviewed) report was retained and the linkage recorded in the extraction log. Narrative reviews were not used as evidentiary sources but were screened for additional primary references.

#### Language restriction

We restricted inclusion to English-language publications for feasibility reasons. We acknowledge that a substantial body of halal-related literature is published in Bahasa Malaysia, Bahasa Indonesia, Arabic, and Turkish, particularly in journals indexed regionally rather than internationally, and that this restriction is a likely source of selection bias. This limitation is discussed under “Limitations”.

### Evidence categorisation and weighting

The four review domains draw on intrinsically heterogeneous bodies of evidence: empirical (analytical and biotechnological), jurisprudential (textual/doctrinal), and regulatory (normative documents and certification practice). Rather than pooling these sources, we categorised each included record into one of three evidence streams (analytical, jurisprudential, or regulatory) and assessed each within its own quality framework (see below). Findings were then triangulated narratively without quantitative weighting; where evidence streams converged, we report this explicitly, and where they diverged, the divergence is foregrounded rather than reconciled. This explicit separation is intended to avoid conflating empirical findings, doctrinal consensus, and regulatory norms, three categories that operate under distinct epistemic rules.

### Data extraction

Two reviewers (R.L., A.M.) independently screened titles and abstracts in Rayyan, with a third reviewer (W.R.) resolving disagreements. Data were extracted using a pre-piloted form covering: study design, country, evidence stream, sample or product type, analytical platform or jurisprudential framework, target species, limit of detection, certification body or fatwa source, and principal findings.

### Risk of bias assessment

Risk of bias in analytical and method-validation studies was assessed with a modified QUADAS-2 tool, adapted to include domains for matrix relevance (native vs hydrolysed), assay specificity, and validation status (in-house vs ISO-validated). Each domain was rated as low, unclear, or high risk by two reviewers independently.

For jurisprudential sources, which fall outside the scope of standard risk-of-bias instruments designed for empirical research, we developed and applied an internal-consistency checklist with four domains: (i) explicit citation of primary jurisprudential sources (Qur'an, hadith collections, classical fiqh manuals); (ii) representation of the four mainstream Sunni schools and, where relevant, of Shi'a positions; (iii) transparency regarding the use of istihalah and darurah doctrines; and (iv) explicit distinction between scholarly opinion, majority position, and binding regulatory norm. Each source was rated low, unclear, or high concern per domain.

For regulatory documents, we recorded the issuing authority, scope (national vs supranational), and binding status.

### Certainty of evidence

Within each evidence stream, we rated overall certainty using a GRADE-informed framework adapted for non-clinical-outcome reviews. For analytical findings, certainty considered consistency across laboratories, validation status, matrix applicability, and reproducibility. For jurisprudential findings, it reflected the breadth of scholarly representation, textual unambiguity, and degree of inter-school agreement, with explicit recognition that doctrinal consensus is not equivalent to empirical certainty. For regulatory findings, it reflected the maturity and inter-jurisdictional convergence of frameworks. Certainty was rated as high, moderate, low, or very low (Table 3).

### Synthesis approach

Given the heterogeneity of evidence types, no quantitative meta-analysis was performed. Results are presented as a structured narrative synthesis organised by domain, with summary tables for jurisprudential classifications, analytical methods, and halal-compliant alternatives. The strength of each principal claim is qualified by its assessed certainty of evidence.

## Results

### Study selection

**Fig. 1:**
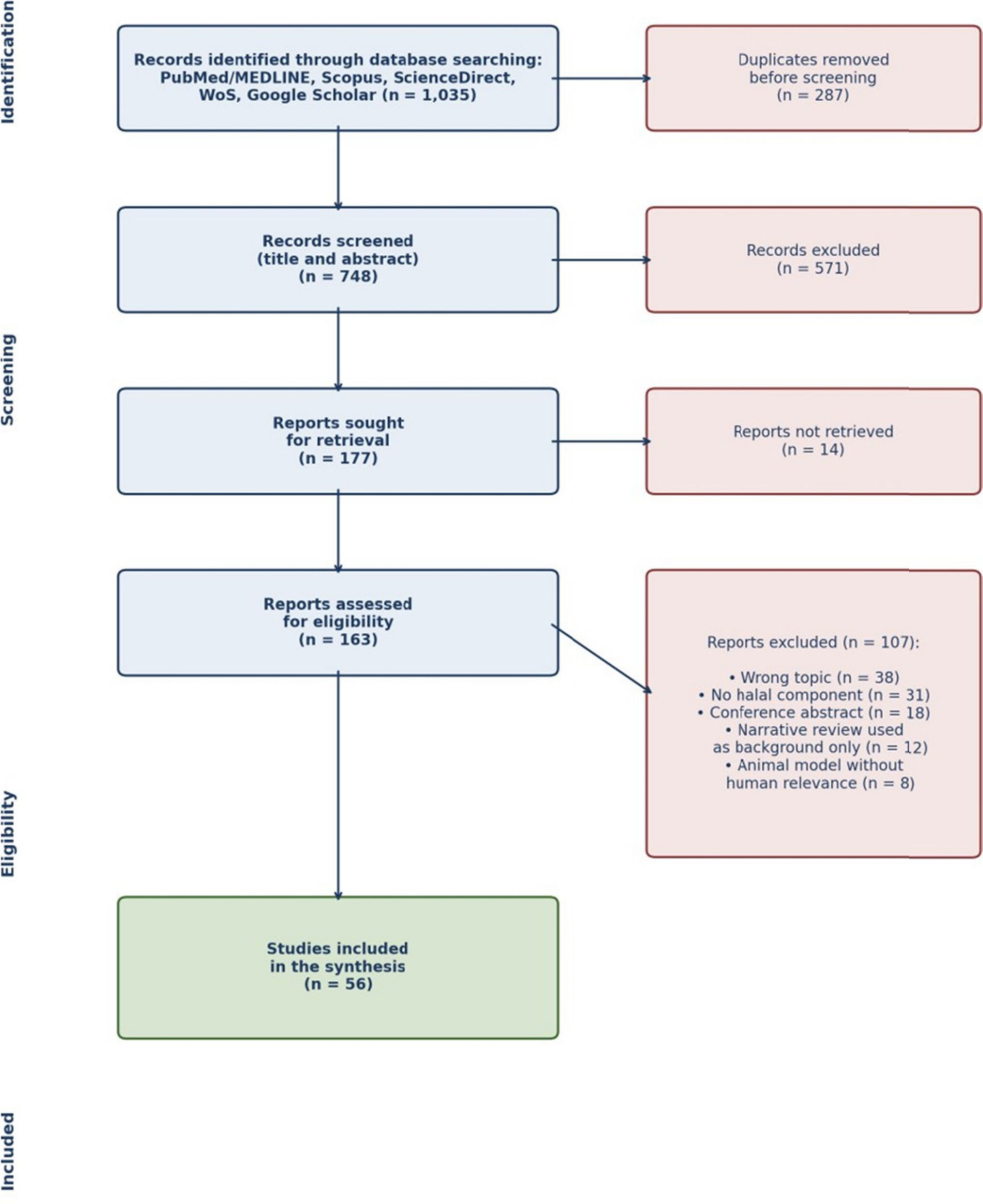
PRISMA 2020 flow diagram of the study selection process

Of 1035 records identified, 287 duplicates were removed prior to screening. Title and abstract screening of 748 records excluded 571; of the remaining 177 reports sought for retrieval, 14 could not be obtained. Full-text assessment of 163 reports led to exclusion of 107 (wrong topic *n* = 38; no halal component *n* = 31; conference abstract *n* = 18; narrative review used as background only *n* = 12; animal model without human relevance *n* = 8). Fifty-six studies were ultimately included (Fig. 1).

### Study characteristics

The 56 included studies span five evidence categories: Islamic jurisprudence/fiqh (*n* = 12), analytical authentication (*n* = 18), halal-compliant alternatives (*n* = 14), pharmaceutical and cosmetic applications (*n* = 9), and market/regulatory dimensions (*n* = 3). Publications ranged from 2001 to 2025, with marked acceleration after 2015. The most represented countries of origin were Malaysia (*n* = 19), Indonesia (*n* = 11), Turkey (*n* = 8), and the UK (*n* = 6). A summary of study characteristics by evidence stream is shown in Table 2.

**Table 2:** Summary of included studies by evidence stream (*n* = 56)

Evidence stream	Number	Predominant study designs	Sample/product types or frameworks	Principal analytical or doctrinal output
Jurisprudential/fiqh	12	Doctrinal analyses; fatwa-council statements; thematic reviews of fiqh	Qur'anic and hadith primary sources; four Sunni schools; selected Shi'a positions	Classification of porcine, bovine, fish, poultry, and recombinant collagen; application of istihalah and darurah
Analytical authentication	18	Method-development and method-validation studies; inter-laboratory comparisons	Pharmaceutical capsules, gelatin powders, collagen supplements; native and hydrolysed matrices	Limits of detection, specificity, and validation status of qPCR, duplex PCR, LC–MS/MS, ELISA, FTIR + PLS-DA, and hydroxyproline assays
Halal-compliant alternatives	14	Process-development studies; comparative characterisation; technology reviews	Fish/marine collagen; Etawa goat skin; plant-based hydrocolloids; recombinant systems (*E. coli*, *P. pastoris*)	Yield, gel strength, denaturation temperature, scalability, and certification feasibility
Pharmaceutical and cosmetic applications	9	Formulation studies; product-survey audits; case reports	Hard and soft capsules; topical formulations; biomedical devices	Prevalence of undeclared porcine collagen; reformulation pathways
Market/regulatory	3	Policy analyses; certification-framework reviews	MS 2424:2019 (Malaysia); IFANCA/ISA (USA); ESMA (UAE); GCC certifying bodies	Scope, binding status, and degree of inter-jurisdictional convergence

### Risk of bias and certainty of evidence

Across the 18 analytical studies, 11 were rated low risk of bias on the modified QUADAS-2, 5 unclear, and 2 high risk (mainly due to incomplete reporting of assay specificity in hydrolysed matrices). Of the 12 jurisprudential sources, 7 were rated low concern on the internal-consistency checklist, 4 unclear (mainly for limited representation of non-dominant scholarly positions), and 1 high concern. Regulatory documents were appraised qualitatively rather than rated. A summary of certainty of evidence for the principal findings is shown in Table 3.

**Table 3:** Certainty of evidence (GRADE-informed) for principal findings, by review domain

Principal finding	Certainty	Main reasons for downgrading (or upgrading)
Porcine collagen is classified as prohibited (haram) across mainstream Sunni schools, independent of industrial processing	High	Convergent textual and doctrinal evidence; high inter-school agreement; very limited variation in scholarly opinion
Bovine collagen is permissible conditional on ritual (zabiha) slaughter and certified traceability	Moderate	Broad agreement in principle, but with variation between schools and certifying bodies on permissibility of non-zabiha bovine and on documentary requirements
Fish collagen is broadly permissible (halal) across mainstream schools	Moderate–high	Convergent doctrinal position; main residual uncertainty concerns cross-contamination in shared manufacturing facilities
qPCR has the most favourable overall profile for native collagen matrices (LOD ≈ 0.01%)	Moderate	Consistent across independent laboratories; downgraded for limited applicability to extensively hydrolysed matrices
LC–MS/MS proteomics is preferable for extensively hydrolysed matrices	Moderate	Convergent evidence from multiple laboratories; downgraded for cost, reproducibility heterogeneity, and false positives from repetitive Gly-X–Y motifs
Fish-derived collagen is the most commercially mature halal alternative	Moderate	Convergent process-development evidence; downgraded for variable gel strength and lower denaturation temperature relative to mammalian collagen
Recombinant collagen offers the highest theoretical halal assurance	Low–moderate	Strong in principle but downgraded for cost, limited regulatory translation, and the requirement that fermentation substrates be themselves halal-certified
International harmonisation of halal pharmaceutical standards represents an unmet regulatory need	Low	Based predominantly on policy analyses and expert interpretation rather than empirical outcome data

Certainty categories adapted from GRADE for non-clinical-outcome reviews

### Islamic jurisprudential framework

#### Qur'anic foundations and the four Sunni schools

The prohibition of swine is grounded in textually unambiguous (qat'i) Qur'anic verses (Al-Baqarah (2:173), Al-Ma'idah (5:3), and Al-An'am (6:145)) and is therefore treated by mainstream Sunni jurisprudence as an inviolable legal norm [10]. The four classical Sunni schools converge on this position in their primary manuals: al-Sarakhsi for the Hanafi school [12], Ibn Rushd al-Hafid for the Maliki school [13], al-Nawawi for the Shafi'i school [14], and Ibn Qudamah for the Hanbali school [15]. This convergence is reflected in the binding Resolution No. 67/3/7 of the International Islamic Fiqh Academy of the OIC (1995) [11] and in the standing position of the European Council for Fatwa and Research [16]. We describe this as a high inter-school agreement rather than as a strictly “unanimous” position, since intra-school nuance and minority dissent can exist in any classical tradition and may not be fully represented in the indexed English-language literature.

Bovine collagen is conditionally permissible, subject to ritual (zabiha) slaughter performed with the invocation (tasmiyah), drainage of blood, and documented traceability [10, 18]. The Hanafi school, drawing on al-Sarakhsi's treatment of dry impurities [12], recognises additional flexibility for dry bone-derived material; the Maliki [13], Shafi'i [14], and Hanbali [15] schools are more conservative on non-zabiha bovine sources. Fish-derived collagen is broadly accepted without the requirement of ritual slaughter, with residual concerns relating chiefly to cross-contamination in mixed-source manufacturing facilities. Source-by-source jurisprudential status is summarised in Table 4.

**Table 4:** Collagen sources and jurisprudential status across mainstream Sunni schools

Source	Estimated global share	Status (mainstream Sunni view)	Notes and intra-tradition variation
Porcine (Sus scrofa)	~ 41%	Haram (high agreement)	Consistent classification across the four mainstream Sunni schools [10, 12–15]; affirmed by IIFA-OIC Resolution 67/3/7 [11] and the ECFR [16]; industrial transformation (istihalah) is not generally accepted as altering this status [17]; minority and case-specific darurah arguments exist but are not used to redefine the underlying classification
Bovine—non-zabiha	~ 28.5%	Haram (majority view)	Classified as haram by the Maliki, Shafi'i, and Hanbali schools; the Hanafi school recognises some flexibility for dry, blood-free bone-derived material
Bovine—zabiha	—	Halal (high agreement)	Requires zabiha slaughter with tasmiyah, full blood drainage, and credible third-party certification; documentary traceability is a frequent point of regulatory variation
Fish (Pisces spp.)	~ 1% (growing)	Halal (high agreement)	No ritual slaughter requirement in mainstream Sunni jurisprudence; cross-contamination risk in shared facilities is the principal residual concern
Poultry (zabiha)	Minor in collagen market	Halal (high agreement)	Type I/III collagen; increasingly used in halal pharmaceutical capsule markets
Recombinant	Emerging	Potentially halal—case-by-case	Permissibility depends on fermentation substrates being themselves halal-certified and on chain-of-custody documentation; no settled cross-jurisdictional position

Statuses reflect the dominant Sunni scholarly view as represented in the included English-language literature; intra-tradition variation, Shi'a positions, and reformist views are not fully represented and warrant dedicated study

We note explicitly that the literature included here is dominated by Sunni jurisprudential sources; Shi'a, Ibadi, and reformist perspectives are under-represented. This is a meaningful limitation for any claim of “consensus”, and we have therefore used the language of majority opinion or high inter-school agreement throughout the manuscript.

#### Istihalah, darurah, and scholarly nuance

The doctrine of istihalah, the transformation of a substance’s essential nature such that its juridical status changes, is sometimes invoked in discussions of gelatin. Its applicability to collagen is contested. The dominant position, supported by IIFA-OIC Resolution 67/3/7 [11] and by the ECFR [16], holds that because collagen retains species-identifying amino acid sequences after standard industrial processing, the source animal remains jurisprudentially determinative [10, 17]. A minority position, anchored in the Hanafi treatment of essential change in al-Sarakhsi [12] and developed in contemporary discussion [17], holds that extensive chemical or enzymatic hydrolysis can in principle satisfy the criteria of istihalah, and some certifying bodies have used this argument in narrow technical contexts. The literature does not yet provide a fully convergent doctrinal answer.

The doctrine of darurah (necessity) permits temporary use of otherwise prohibited materials when no halal alternative is available, a qualified clinician confirms necessity, and use is minimised in quantity and duration [10, 18]. The Malaysian Fatwa Council has invoked darurah to permit administration of a polio vaccine containing porcine-derived trypsin in the absence of viable alternatives [17]. The threshold for invoking darurah, and the question of whether commercial availability of an alternative in one jurisdiction defeats a darurah argument in another, varies across scholarly authorities and certifying bodies [11, 16], and is itself a matter of active doctrinal discussion.

### Analytical authentication

Eighteen analytical studies addressed species-level authentication of collagen or gelatin in pharmaceutical, cosmetic, or food matrices. The most prevalent platforms were quantitative PCR (qPCR) targeting mitochondrial DNA, duplex PCR for simultaneous bovine and porcine detection, LC-MS/MS proteomics using species-specific peptide biomarkers, ELISA with anti-porcine antibodies, FTIR coupled with partial least squares discriminant analysis (PLS-DA), and hydroxyproline quantification. A comparative summary, expanded to include specificity, validation status, and typical false-positive/false-negative scenarios, is shown in Table 5.

**Table 5:** Analytical methods for halal authentication of collagen and gelatin: principle, performance, validation, and failure modes

Method	Principle	LOD	Specificity/validation status	Typical false-positive scenarios	Typical false-negative scenarios
qPCR (mtDNA)	Real-time amplification of species-specific mitochondrial DNA targets	≈ 0.01%	High species specificity when primer/probe design is validated; ISO-validated kits available [19, 20]	Cross-amplification with closely related species if primers are poorly designed; carry-over contamination in shared facilities	Extensively hydrolysed matrices in which DNA is degraded below the amplifiable length [21]
Duplex PCR	Simultaneous amplification of bovine and porcine mtDNA targets	≈ 0.1%	Validated in pharmaceutical capsule matrices [19]; lower analytical sensitivity than qPCR	Primer-dimer artefacts mis-identified as positive signals	DNA degradation in heat-processed products; competitive inhibition between targets
LC–MS/MS proteomics	Detection of species-specific tryptic peptide biomarkers	≈ 0.1%	High specificity when biomarker peptides are bioinformatically curated and orthogonally validated [21]; reproducibility heterogeneous across laboratories	Cross-reactivity from repetitive Gly-X–Y motifs shared across collagen homologues; carry-over in shared LC systems	Low-abundance porcine contamination falling below proteome-level detection thresholds
ELISA	Antibody recognition of porcine collagen epitopes	≈ 0.033 mg/mL	Validated in selected food and supplement matrices [22, 23]; sensitivity lower than nucleic-acid methods	Antibody cross-reaction with bovine collagen epitopes	Epitope destruction by heat or chemical processing
FTIR + PLS-DA	Infrared spectral fingerprinting combined with multivariate classification	Semi-quantitative	Useful as a rapid screening tool; species-level specificity insufficient as a stand-alone confirmatory method	Misclassification in mixed-source samples not represented in the training set	Overlapping spectral features between species at low contamination levels
Hydroxyproline assay	Quantification of a collagen-signature amino acid (ISO 17025)	Qualitative	Robust and inter-laboratory reproducible [24]; does not discriminate between animal species	Not applicable—does not provide species-level identification	Not applicable—does not provide species-level identification

*LOD* limit of detection, *LC–MS/MS* liquid chromatography–tandem mass spectrometry, *FTIR* Fourier-transform infrared spectroscopy, *PLS-DA* partial least squares discriminant analysis

#### Why qPCR—but not as a stand-alone gold standard

qPCR targeting mitochondrial DNA shows the most favourable overall profile for native collagen matrices in the included literature: limits of detection in the order of 0.01% [19, 20], well-characterised specificity when primer and probe design is properly validated, ISO-validated commercial kits, and short turnaround time. However, in extensively hydrolysed matrices DNA is degraded to fragment lengths below those amplifiable by standard qPCR assays, and method performance drops accordingly [21]. LC-MS/MS proteomics retains species-level specificity in such matrices but at higher cost and with greater inter-laboratory variability. ELISA provides an independent, protein-based readout with a different error profile from PCR-based methods. We therefore describe qPCR as the most favourable single method for native matrices rather than as an absolute gold standard, and recommend orthogonal testing (typically qPCR with ELISA, and LC-MS/MS for hydrolysed products) where the stakes warrant confirmation [22, 23]. Hydroxyproline quantification remains a useful preliminary screening marker but cannot discriminate species [24]. A recent study reported detection of porcine DNA in products labelled as marine-derived, highlighting cross-contamination risks in shared manufacturing facilities and the value of routine orthogonal authentication in supply-chain quality assurance [23].

### Halal-compliant alternatives

Four categories of halal-compliant alternatives were identified across the 14 included studies in this stream: halal animal-based collagen (predominantly fish and goat); plant-based hydrocolloids functioning as collagen substitutes in selected applications; microbial polymers; and recombinant collagen. Each was assessed along four distinct axes: technical feasibility, commercial scalability, regulatory approval status, and economic practicality, that the existing literature does not always distinguish (Table 6).

**Table 6:** Halal-compliant alternatives to porcine collagen, separated by technical feasibility, commercial scalability, regulatory approval, and economic practicality

Alternative	Status	Technical feasibility	Commercial scalability	Regulatory approval	Economic practicality
Halal animal-based (fish; Etawa goat)	Halal (high agreement)	High—native type I collagen; bioavailability comparable to porcine [25, 26, 28]	High for fish-derived; moderate for goat-derived	Mature pathways in Muslim-majority jurisdictions; established in MS 2424:2019 [30]	Cost premium over porcine but commercially competitive at scale
Plant-based hydrocolloids (agar, carrageenan, pectin, starch–alginate)	Halal	Partial—cannot reproduce triple-helix function; substitutes only in selected applications	High	Well established as pharmaceutical excipients	Generally favourable; cost-competitive
Microbial polymers (xanthan, chitosan)	Halal if substrate halal	Moderate—application-specific functional overlap with collagen	High	Established; halal status contingent on substrate certification	Generally favourable
Recombinant collagen (*E. coli*, *P. pastoris*, plant systems)	Potentially halal—case-by-case	High—sequence-precise; BSE-free [27, 29]	Currently moderate; scale-up pathways exist but are not yet widely commercial	Limited; no harmonised cross-jurisdictional approval framework yet [29]	High cost; economic barrier to broad pharmaceutical translation

*BSE* bovine spongiform encephalopathy

Recombinant systems based on *E. coli*, *Pichia pastoris*, and engineered plant hosts offer the highest theoretical halal assurance, are intrinsically free of zoonotic risk, and enable sequence-precise production [27, 29]. Their clinical translation is constrained by three distinct barriers that the literature does not always separate. *First*, fermentation substrates (culture media, growth factors) must themselves be halal-certified, and chain-of-custody documentation is not always available. *Second*, regulatory approval pathways for recombinant collagen as a pharmaceutical excipient are still maturing and are not yet harmonised across jurisdictions [29]. *Third*, cost remains substantially higher than that of conventional collagen sources, which limits broad pharmaceutical adoption. These three constraints operate on different time horizons and should not be conflated when evaluating the feasibility of recombinant alternatives.

### Regulatory dimensions

Malaysia’s MS 2424:2019 is the most comprehensive national halal pharmaceutical standard identified in the literature, with approximately 1200 JAKIM-certified products in 2024 [30]. Internationally, fragmentation is substantial: IFANCA and ISA in the USA require zabiha bovine sources; ESMA in the United Arab Emirates applies stricter criteria; and selected GCC certifying bodies conditionally accept non-zabiha bovine [30]. Hassan et al. reported that Muslim patients unaware of the halal status of their medication were more likely to self-modify therapy without physician consultation [6]; this finding is suggestive of an adherence-related compliance risk, although the dataset was not rheumatology-specific.

## Discussion

This review yields three principal observations, each qualified by its assessed certainty of evidence. First, porcine collagen is classified as prohibited with high inter-school agreement and is not generally rendered permissible by standard industrial processing [10–17]; undisclosed porcine excipients therefore raise a recurrent disclosure issue for Muslim patients. Second, qPCR combined with ELISA (supplemented by LC-MS/MS in extensively hydrolysed matrices) offers a robust orthogonal authentication strategy suitable for pharmaceutical supply-chain quality assurance [19–23]. Third, fish-derived collagen is the most commercially mature halal-compliant alternative, while recombinant systems offer the highest theoretical halal assurance but face cost, regulatory, and substrate-certification barriers [25–29].

### Distinguishing consensus, majority opinion, and regulatory practice

We have avoided the language of strict “consensus” where the evidence supports only a majority position. The porcine classification is robust across the included Sunni sources [10–17]; the bovine classification is more variable, with scholarly disagreement on the documentary requirements for zabiha traceability; the classification of recombinant collagen remains unsettled. Regulatory practice is an independent stratum: a rule in one jurisdiction does not constitute scholarly consensus across the broader tradition.

### Relevance to rheumatology—and the limits of extrapolation

Direct rheumatology-specific evidence is limited. The vaccination-hesitancy data of Leone et al. [7] and the medication-modification data of Hassan et al. [6] are suggestive of an adherence pathway by which concern over animal-derived excipients may affect long-term therapy, but neither study was conducted in a rheumatology cohort. Leone’s medical-anthropological framework [8] offers a conceptual rationale for proactive halal counselling in the prescription of biologic agents, gelatin-containing capsules, and collagen-based devices, but we frame this as an extrapolation rather than a rheumatology-specific recommendation. Dedicated cohort studies are required before stronger claims can be supported.

### Disentangling alternatives

The four-axis presentation in Table 6 addresses a recurrent ambiguity in the literature, where technical feasibility, commercial scalability, regulatory approval, and economic practicality are sometimes conflated. Fish collagen is mature on all four axes; recombinant collagen is technically high-performing but commercially nascent, regulatorily uneven, and economically constrained. Progress on one axis does not imply progress on the others.

### What this review is and is not

Several conclusions are best understood as evidence-based extrapolations rather than direct empirical findings, in particular those relating to pharmaceutical quality-control implementation and international regulatory harmonisation. In the “Conclusions”, we separate statements supported by high or moderate certainty from reasoned expert interpretation of policy gaps.

### Limitations

Several limitations must be acknowledged. *First*, the restriction to English-language sources is likely to have under-represented a substantial body of regionally indexed literature in Bahasa Malaysia, Bahasa Indonesia, Arabic, and Turkish, and our characterisation of “high inter-school agreement” should be read in this light. *Second*, the geographic distribution of analytical and process-development studies is concentrated in Southeast Asia, which may limit generalisability. *Third*, sample sizes in several authentication studies are modest, particularly for hydrolysed pharmaceutical matrices. *Fourth*, direct rheumatology-specific evidence on clinical outcomes of halal-related medication concerns is sparse, and the present synthesis relies on indirect extrapolation from adjacent fields such as vaccination uptake. *Fifth*, standard risk-of-bias instruments are not designed for jurisprudential sources; we addressed this by developing and reporting an internal-consistency checklist, but this tool has not undergone formal external validation. *Sixth*, Shi'a, Ibadi, and reformist scholarly positions are under-represented in the included literature, and any future revision of this synthesis would benefit from systematic inclusion of these traditions and from continued consultation with experts in Islamic pharmaceutical jurisprudence.

## Conclusions

Within the limits of the available English-language literature, this PRISMA 2020-compliant review supports four broad observations. In mainstream Sunni jurisprudence, porcine collagen is classified as prohibited with high inter-school agreement and is not generally rendered permissible by standard industrial processing; bovine collagen is conditionally permissible subject to ritual slaughter and certified traceability, with some scholarly variation; fish collagen is broadly accepted and is the most commercially mature halal-compliant alternative. Orthogonal analytical authentication (qPCR with ELISA for native matrices and LC-MS/MS for extensively hydrolysed products) warrants consideration in pharmaceutical supply-chain quality assurance. Recombinant collagen is a technically promising option whose translation depends on the separable axes of substrate certification, regulatory approval, and cost reduction. The international harmonisation of halal pharmaceutical standards is, on the basis of the present evidence, a plausible regulatory priority rather than an established empirical conclusion.

For clinicians serving Muslim patients, including, but not limited to, rheumatologists prescribing biologic agents, gelatin-containing oral therapies, and collagen-based medical devices, these findings support proactive discussion of halal status in patient counselling, while recognising that direct rheumatology-specific outcome evidence remains an open research question. Dedicated cohort studies of adherence and outcomes in Muslim rheumatology populations would substantially strengthen the evidence base on which such counselling rests.

## Supplementary Information

Below is the link to the electronic supplementary material.

ESM 1 Supplementary Material 1: Internal review protocol and any amendments. (DOCX 26.0 KB)

ESM 2 Supplementary Material 2: Full database-specific search strings, hit counts, and screening logs. (DOCX 26.6 KB)

## Data Availability

The complete search strategy, screening logs, data-extraction tables, risk-of-bias matrices, and certainty-of-evidence judgments are available from the corresponding author on reasonable request (see also Supplementary Material S1- S2).

## Declarations

### Protocol registration

This review was not prospectively registered in a public systematic-review registry. The protocol, search logs, data-extraction matrices, risk-of-bias judgments, and certainty-of-evidence ratings are available from the corresponding author on reasonable request.

### Interdisciplinary methodological note

The jurisprudential domain of this review was constructed by reading primary sources (Qur'an; classical fiqh manuals of the four Sunni schools; binding resolutions of the IIFA-OIC; standing position of the European Council for Fatwa and Research) alongside the secondary literature. Where the doctrinal record diverges, we have foregrounded the divergence rather than reconciled it. We acknowledge that a formal consultation with a specialist in Islamic pharmaceutical jurisprudence would further strengthen the doctrinal accuracy of any future iteration of this work; this is identified as a research priority in the “Limitations” section. To this end, the authors are committed to securing formal consultation with a specialist in Islamic pharmaceutical jurisprudence and halal regulation in any subsequent revision or primary study arising from this work; the doctrinal classifications reported here should accordingly be read as the dominant scholarly view as represented in the included literature, rather than as definitive juridical rulings.
