# Supplementary material for: Animal-derived collagen and Islamic religious prescriptions: a systematic review
Source: Clin Rheumatol. 2026 Jun 25;45(8):4801–12. doi: 10.1007/s10067-026-08247-z (PMC13486105; doi:10.1007/s10067-026-08247-z)
Supplement: Supplementary file 1 [file 10067_2026_8247_MOESM1_ESM.docx]

**Supplementary Material S1**

**Review Protocol: Animal-Derived Collagen and Islamic Religious Prescriptions: A Systematic Review**

# 1. Background and Rationale

Collagen, the most abundant structural protein in vertebrates, is widely used as a pharmaceutical, cosmetic, and biomedical excipient. Approximately 41% of global collagen and gelatin production is of porcine origin, creating a recurrent conflict with Islamic dietary law for the estimated 1.9 billion Muslim patients worldwide. No prior synthesis had integrated jurisprudential, analytical, and regulatory evidence within a single PRISMA 2020-compliant review oriented to rheumatology and pharmaceutical practice. The present protocol was developed to address that gap.

# 2. Review Question

What does the available peer-reviewed and primary jurisprudential literature show regarding (i) the Islamic jurisprudential classification of collagen by animal source; (ii) validated analytical methods for halal authentication of collagen and gelatin; (iii) halal-compliant alternatives to porcine collagen; and (iv) the international regulatory landscape for halal pharmaceuticals (and what implications, if any, can be cautiously extrapolated to clinical practice in rheumatology?).

# 3. Objectives

• Objective 1: Jurisprudential. To synthesise the classification of collagen by animal source across mainstream Sunni schools, distinguishing scholarly consensus, majority opinion, and regulatory practice; to characterise the contemporary application of istihalah and darurah doctrines.

• Objective 2: Analytical. To synthesise validated analytical methods for species-level authentication of collagen and gelatin, with explicit attention to specificity, validation status, and applicability across native and hydrolysed product matrices.

• Objective 3: Alternatives. To characterise halal-compliant alternative collagen sources along four explicit axes: technical feasibility, commercial scalability, regulatory approval, and economic practicality.

• Objective 4: Regulatory. To describe the international landscape of halal pharmaceutical certification frameworks and to identify the principal gaps relevant to clinical and pharmaceutical practice in rheumatology and adjacent fields.

# 4. Eligibility Criteria

## 4.1 Inclusion

• Peer-reviewed original research, systematic reviews, and method-validation studies.

• Identifiable jurisprudential analyses, including primary fiqh sources, binding fatwa-council resolutions, and peer-reviewed doctrinal studies.

• Records addressing at least one of the four review domains.

• Publications between 1 January 2000 and 31 March 2026.

• Accessible full text in English.

## 4.2 Exclusion

• Conference abstracts without full text.

• Editorial pieces without primary data or structured argumentation.

• Purely physicochemical studies without a halal component.

• Animal-only studies without explicit human-relevance discussion.

• Narrative reviews used only as background — primary references checked but the review itself not retained as an evidentiary source.

• Duplicate publications or reports drawing on overlapping primary datasets, the most complete (typically most recent peer-reviewed) report retained, and the linkage recorded in the extraction log.

## 4.3 Language Restriction

Inclusion was restricted to English-language publications for feasibility. We explicitly acknowledge that a substantial body of halal-related literature is published in Bahasa Malaysia, Bahasa Indonesia, Arabic, and Turkish, particularly in regionally indexed journals, and that this restriction is a likely source of selection bias. This is reported as a limitation in the main manuscript.

# 5. Information Sources and Search Strategy

Five bibliographic databases were searched between 1 February and 31 March 2026: PubMed/MEDLINE, Scopus, ScienceDirect, Web of Science Core Collection, and Google Scholar (first 200 records by relevance). Reference lists of included studies and of three recent reviews were hand-searched. Records were managed in EndNote 21. The complete database-specific search strings, controlled-vocabulary terms (MeSH for PubMed; Emtree for Scopus), Boolean operators, field tags, applied filters, and per-database hit counts are reported in Supplementary Material S2 and reproduced in Table 1 of the main manuscript.

# 6. Evidence Categorisation and Weighting

The four review domains draw on intrinsically heterogeneous evidence: empirical (analytical, biotechnological), jurisprudential (textual and doctrinal), and regulatory (normative documents, certification practice). Rather than pooling sources, each included record was assigned to one of three evidence streams (analytical, jurisprudential, regulatory) and appraised within its own quality framework. Findings were then triangulated narratively without quantitative weighting; convergence was reported explicitly, divergence foregrounded rather than reconciled. This explicit separation is intended to prevent conflation of empirical findings, doctrinal consensus, and regulatory norms. three categories that operate under distinct epistemic rules.

# 7. Study Selection and Data Extraction

Two reviewers (R.L. A.M) independently screened titles and abstracts in Rayyan; disagreements were resolved by a third reviewer (W.R.). A pre-piloted data-extraction form was used, covering: study design, country, evidence stream, sample or product type, analytical platform or jurisprudential framework, target species, limit of detection, certification body or fatwa source, and principal findings.

# 8. Risk-of-Bias Assessment

Analytical and method-validation studies were assessed with a modified QUADAS-2, adapted to include three additional domains: matrix relevance (native vs hydrolysed), assay specificity, and validation status (in-house vs ISO-validated). Each domain was rated as low, unclear, or high risk by two reviewers independently.

Jurisprudential sources, which fall outside the scope of standard risk-of-bias instruments designed for empirical research, were assessed with an internal-consistency checklist comprising four domains: (i) explicit citation of primary jurisprudential sources (Qur'an, hadith collections, classical fiqh manuals); (ii) representation of the four mainstream Sunni schools and, where relevant, of Shi'a positions; (iii) transparency regarding the use of istihalah and darurah doctrines; and (iv) explicit distinction between scholarly opinion, majority position, and binding regulatory norm. Each source was rated as low, unclear, or high concern per domain. This checklist has not undergone formal external validation; this is acknowledged in the manuscript Limitations.

Regulatory documents were appraised qualitatively rather than rated, recording the issuing authority, scope (national vs supranational), and binding status.

# 9. Certainty of Evidence

Within each evidence stream, overall certainty of evidence was rated using a GRADE-informed framework adapted for non-clinical-outcome reviews. For analytical findings, certainty considered consistency across independent laboratories, validation status, applicability to relevant product matrices, and reproducibility. For jurisprudential findings, certainty reflected the breadth of scholarly representation, the unambiguity of the underlying textual sources, and the degree of inter-school agreement, with explicit recognition that doctrinal consensus is not equivalent to empirical certainty. For regulatory findings, certainty reflected the maturity and inter-jurisdictional convergence of frameworks. Certainty was rated as high, moderate, low, or very low.

# 10. Synthesis Approach

Given the heterogeneity of evidence types, no quantitative meta-analysis was planned or performed. Results are presented as a structured narrative synthesis organised by domain, with summary tables for jurisprudential classifications, analytical methods, and halal-compliant alternatives. The strength of each principal claim is qualified by its assessed certainty of evidence.

# 11. Registration

This review was not prospectively registered in a public systematic-review registry such as PROSPERO. We acknowledge that prospective registration represents methodological best practice. The present protocol, the search logs and hit counts, the screening decisions, the data-extraction matrices, and the risk-of-bias judgments are documented and are available from the corresponding author on reasonable request to support reproducibility of the synthesis.

# 12. Protocol Amendments

The protocol underwent two amendments during the review, documented here for transparency:

• Amendment 1: Data-extraction template refined after pilot screening of the first 20 full-text records, to harmonise extraction across analytical and jurisprudential streams (added field: 'certification body or fatwa source').

• Amendment 2: Certainty-of-evidence assessment (GRADE-informed framework) added after completion of risk-of-bias assessment, in response to peer-review feedback emphasising the need for formal evidence grading. This amendment did not affect study selection or data extraction.
