# Supplementary material for: Animal-derived collagen and Islamic religious prescriptions: a systematic review
Source: Clin Rheumatol. 2026 Jun 25;45(8):4801–12. doi: 10.1007/s10067-026-08247-z (PMC13486105; doi:10.1007/s10067-026-08247-z)
Supplement: Supplementary file 2 [file 10067_2026_8247_MOESM2_ESM.docx]

**Supplementary Material S2**

**Search Strategy and Screening Logs — Animal-Derived Collagen and Islamic Religious Prescriptions: A Systematic Review**

# 1. Overview

Five bibliographic databases were searched between 1 February and 31 March 2026: PubMed/MEDLINE, Scopus, ScienceDirect, Web of Science Core Collection, and Google Scholar (first 200 records by relevance). Reference lists of included studies and of three recent reviews were hand-searched. All retrieved records were managed in EndNote 21. Duplicates were removed with a two-step procedure: automated identifier matching (DOI / PubMed ID / title), followed by manual review of near-duplicate titles. Searches were limited to records published between 1 January 2000 and 31 March 2026; no methodological filters were applied at the search stage.

# 2. Database-Specific Search Strings (Verbatim)

## 2.1 PubMed/MEDLINE

Search performed on 5 February 2026 via <https://pubmed.ncbi.nlm.nih.gov/>.

("Collagen"[MeSH] OR "Gelatin"[MeSH] OR collagen[tiab] OR gelatin*[tiab]) AND ("Islam"[MeSH] OR halal[tiab] OR haram[tiab] OR "Islamic law"[tiab] OR shari'a[tiab] OR istihalah[tiab] OR darurah[tiab] OR "religious dietary law"[tiab]) AND (porcine[tiab] OR pork[tiab] OR swine[MeSH] OR bovine[tiab] OR fish[tiab] OR marine[tiab] OR recombinant[tiab] OR authentication[tiab] OR PCR[tiab] OR ELISA[tiab] OR "mass spectrometry"[tiab])

**Filters applied:** Language: English; Publication date: 2000/01/01 – 2026/03/31. No species filter (humans not enforced).

## 2.2 Scopus

Search performed on 8 February 2026 via https://www.scopus.com.

TITLE-ABS-KEY((collagen OR gelatin*) AND (halal OR haram OR islam* OR shari'a OR istihalah OR darurah) AND (porcine OR pork OR swine OR bovine OR fish OR marine OR recombinant OR authentication OR PCR OR ELISA OR "mass spectrometry")) AND PUBYEAR > 1999 AND LANGUAGE(english)

**Filters applied:** Document type: article, review, conference paper with full text; Language: English; PUBYEAR > 1999.

## 2.3 ScienceDirect

Search performed on 12 February 2026 via https://www.sciencedirect.com.

(collagen OR gelatin) AND (halal OR islam* OR "porcine detection" OR "species authentication") AND (pharmaceutical OR cosmetic OR capsule OR excipient)

**Filters applied:** Article type: Research articles, Review articles; Year: 2000–2026.

## 2.4 Web of Science Core Collection

Search performed on 18 February 2026 via Clarivate Web of Science.

TS=((collagen OR gelatin) AND (halal OR haram OR islam* OR shari'a OR istihalah OR darurah) AND (porcine OR bovine OR fish OR marine OR recombinant OR authentication OR PCR OR ELISA OR "mass spectrometry"))

**Filters applied:** Document type: Article, Review; Language: English; Year: 2000–2026; Indexes: SCI-Expanded, SSCI.

## 2.5 Google Scholar

Search performed on 24 February 2026 via https://scholar.google.com.

(collagen OR gelatin) AND (halal OR "islamic dietary law") AND (porcine OR bovine OR fish OR recombinant OR authentication)

**Filters applied:** First 200 results by relevance; Year: 2000–2026. Citation tracking not used.

# 3. Hit Counts per Database

*Table S2.1 Number of records retrieved per database before and after deduplication.*

| **Database** | **Records retrieved** | **After in-database deduplication** | **Date of last search** |
| --- | --- | --- | --- |
| PubMed/MEDLINE | 264 | 262 | 5 February 2026 |
| Scopus | 311 | 308 | 8 February 2026 |
| ScienceDirect | 187 | 186 | 12 February 2026 |
| Web of Science Core Collection | 247 | 243 | 18 February 2026 |
| Google Scholar (top 200 by relevance) | 200 | 200 | 24 February 2026 |
| Hand-search of reference lists | 36 | 36 | 1–15 March 2026 |
| Total before cross-database deduplication | 1,245 | 1,235 | — |
| After cross-database deduplication | — | 1,035 | 16 March 2026 |

*In-database deduplication accounts for indexed duplicates (e.g. preprint + published version with same DOI). Cross-database deduplication accounts for the same record indexed by multiple databases.*

# 4. Screening Log

*Table S2.2 PRISMA 2020-aligned screening log.*

| **Stage** | **n** | **Notes** |
| --- | --- | --- |
| Records identified through database searching | 1,035 | Post cross-database deduplication; matches Fig. 1 of the main manuscript |
| Records identified through hand-searching | (included above) | Reference-list hand-searching contributed 36 records, all retained at screening |
| Total records screened (title and abstract) | 748 | After internal deduplication of additional near-duplicate titles (n = 287 removed) |
| Records excluded at title/abstract stage | 571 | Off-topic; no halal component; no collagen/gelatin focus |
| Reports sought for retrieval | 177 |  |
| Reports not retrieved | 14 | Full text unavailable through institutional access or interlibrary loan |
| Reports assessed for eligibility | 163 |  |
| Reports excluded after full-text review | 107 | See breakdown in Table S2.3 |
| Studies included in the synthesis | 56 | Final n; characteristics summarised in Table 2 of the main manuscript |

# 5. Reasons for Exclusion at Full-Text Stage

*Table S2.3 Reasons for exclusion of full-text records (n = 107).*

| **Reason for exclusion** | **n** | **Notes** |
| --- | --- | --- |
| Wrong topic (collagen/gelatin not the primary focus, or no halal/Islamic angle) | 38 | Most frequent reason |
| No halal component despite topical keyword match | 31 |  |
| Conference abstract without retrievable full text | 18 |  |
| Narrative review used only as background; primary references checked separately | 12 | Reference lists screened for additional primary sources |
| Animal-model study without explicit human-relevance discussion | 8 |  |
| Total | 107 |  |

# 6. Inter-Rater Agreement at Screening

Title-and-abstract screening: percent agreement between R.L. and A.M. was 92.4% (691 of 748 records). The 57 discrepant records were resolved by third-reviewer (W.R.) adjudication, with 21 retained for full-text review and 36 excluded. Full-text screening: percent agreement was 95.1% (155 of 163 records); the 8 discrepant records were resolved by consensus discussion among all three reviewers.

# 7. Software and Data Management

All retrieved records were exported in RIS format and imported into EndNote 21. Title-and-abstract screening was conducted in Rayyan (https://www.rayyan.ai/). Full-text screening was conducted using a shared spreadsheet with one row per record and columns for each inclusion/exclusion criterion. The reviewers' individual screening decisions and adjudication records are retained and are available from the corresponding author on reasonable request.
